# Thyroid dysfunction in Chinese nasopharyngeal carcinoma after anti-PD-1 therapy and its association with treatment response

**DOI:** 10.1186/s12916-022-02697-3

**Published:** 2023-01-16

**Authors:** Zi-Hang Chen, Wei-Hong Zheng, Chen-Fei Wu, Jia Kou, Xing-Li Yang, Li Lin, Jia-Wei Lv, Ying Sun, Guan-Qun Zhou

**Affiliations:** 1 Department of Radiation Oncology, Sun Yat-sen University Cancer Center; State Key Laboratory of Oncology in South China; Collaborative Innovation Center for Cancer Medicine; Guangdong Key Laboratory of Nasopharyngeal Carcinoma Diagnosis and Therapy, 651 Dongfeng Road East, Guangzhou, 510060 People’s Republic of China; 2 Zhongshan School of Medicine, Sun Yat-sen University, Guangzhou, People’s Republic of China

**Keywords:** Anti-PD-1 immunotherapy, Nasopharyngeal carcinoma, Thyroid dysfunction

## Abstract

**Background:**

Programmed cell death protein-1 (PD-1) blockade therapies have demonstrated efficacy in nasopharyngeal carcinoma (NPC). Thyroid dysfunction is among the most common immune-related adverse events. This study aimed to explore the clinical pattern of thyroid dysfunction and its relationship with survival marker in nonmetastatic NPC after immunotherapy.

**Methods:**

From January 1, 2019, to December 31, 2021, 165 pairs of nonmetastatic NPC patients (165 with and 165 without anti-PD-1 immunotherapy) matched by the propensity score matching method were included in this study. Thyroid function was assessed retrospectively before the first treatment and during each immunotherapy cycle.

**Results:**

The spectrum of thyroid dysfunction was different between the immunotherapy and control groups (*P* < 0.001). Compared with the control group, patients in the immunotherapy group developed more hypothyroidism (14.545% vs. 7.273%), less hyperthyroidism (10.909% vs. 23.636%), and a distinct pattern, biphasic thyroid dysfunction (3.030% vs. 0%). Immunotherapy also accelerates the onset of hypothyroidism, which was earlier with a median onset time difference of 32 days (*P* < 0.001). Patients who acquired thyroid dysfunction during immunotherapy had better complete biological response to treatment (OR, 10.980; *P* = 0.042).

**Conclusions:**

For nonmetastatic NPC, thyroid dysfunction was associated with better response to treatment in immunotherapy but not in routine treatment. Thyroid function could be used as a predictor for survival and should be under regular and intensive surveillance in clinical practice of anti-PD-1 immunotherapy for nonmetastatic NPC.

**Supplementary Information:**

The online version contains supplementary material available at 10.1186/s12916-022-02697-3.

## Background

Nasopharyngeal carcinoma (NPC) is especially endemic in Southern China and Southeast Asia, where the age-standardized incidence is as high as 21 per 100,000 person-years [1, 2]. In recent decades, the adoption of intensity-modulated radiotherapy (IMRT) and the widespread use of concurrent chemotherapy have substantially improved the prognosis of NPC [3]. Nevertheless, NPC remains an important cause of cancer-related death, with a global incidence of approximately 50,000 deaths annually [4].

Immune evasion is a well-researched mechanism of carcinogenesis, and anti-PD-1 monoclonal antibodies have shown efficacy in improving the survival of recurrent or metastatic NPC [5–7]. In studies involving anti-PD-1 immunotherapy, one of the most common immune-related adverse events (irAEs) was thyroid dysfunction [8–13]. And the onset of immune-related thyroid dysfunction is found to be associated with significant improvement in clinical outcomes in lung cancer and melanoma [14–16]. Early-phase clinical studies using anti-PD-1 immune checkpoint inhibitors in recurrent or metastatic NPC have reported incidence rates of hypothyroidism during immunotherapy, which were 18.5% for pembrolizumab [5], 6.7% for nivolumab [6], and 32% for camrelizumab [7]. Cuzzocrea et al. reported that a metastatic NPC patient had hypothyroidism and a concomitant rise in anti-thyroid peroxidase (A-TPO) antibody after treatment with nivolumab and chemoradiotherapy [17]. Currently, anti-PD-1 monoclonal antibodies are also used in clinical practice to improve the prognosis of newly diagnosed, nonmetastatic NPC. And the performance status, clinical characteristics, combined therapeutic protocol, and clinical outcome of newly diagnosed, nonmetastatic NPC patients are very different from those of recurrent or metastatic NPC patients. Considering the increasing use of anti-PD-1 immunotherapy in nonmetastatic NPC patients, this study aims to illustrate the incidence and clinical course of immune-related thyroid dysfunction and its role as a predictor of survival in nonmetastatic NPC.

## Methods

### Patients

The medical records of 4003 nonmetastatic NPC patients diagnosed between January 1, 2019, and December 31, 2021, were extracted from the Big Data Intelligence Framework at the Sun Yat-sen University Cancer Centre. The following inclusion criteria were applied: histologically diagnosed NPC based on the World Health Organization criteria and completed chemoradiotherapy (CCRT) or radiotherapy (RT). Among the patients, 786 received at least one dose of PD-1 antibody (i.e., camrelizumab, nivolumab, pembrolizumab, sintilimab, tislelizumab, or toripalimab) during treatment with induction chemotherapy (IC), concurrent chemoradiotherapy (CCRT) or radiotherapy, and 3217 received only IC+CCRT, CCRT or IC+RT. The following exclusion criteria were applied: no normal thyroid function tests within 1 month before treatment; no available thyroid function tests within 2 months after the completion of CCRT or RT; thyroid disease history before treatment; and other malignancies. After review, 165 patients with immunotherapy and 642 patients without immunotherapy were selected. Propensity score matching (PSM) was then used to match the patients who received immunotherapy and the patients who did not receive immunotherapy. Finally, we included 165 patients in the immunotherapy group and 165 patients in the control group. All covariates between the two groups achieved an adequate balance by PSM (Table 1). Additional file 1: Figure S1 shows the patient selection process.

**Table 1:** Distribution of demographic and clinical characteristics of patients in the control and immunotherapy groups

Characteristic	All patients	Control	Immunotherapy	*P*
*n*	330	165	165	
Gender, no. (%)				0.899
Male	246 (74.545)	124 (75.152)	122 (73.939)	
Female	84 (25.455)	41 (24.848)	43 (26.061)	
Age (median (range))	45 (13–71)	45 (13–71)	44 (14–71)	1
WHO histology, no. (%)				1
II	3 (0.909)	1 (0.606)	2 (1.212)	
III	327 (99.091)	164 (99.394)	163 (98.788)	
T stage^a^, no. (%)				0.678
T1	13 (3.939)	5 (3.030)	8 (4.849)	
T2	31 (9.395)	17 (10.303)	14 (8.485)	
T3	178 (53.939)	92 (55.758)	86 (52.121)	
T4	108 (32.727)	51 (30.909)	57 (34.545)	
N stage^a^, no. (%)				0.492
N0	10 (3.030)	3 (1.818)	7 (4.242)	
N1	88 (26.667)	48 (29.091)	40 (24.243)	
N2	103 (31.212)	51 (30.909)	52 (31.515)	
N3	129 (39.091)	63 (38.182)	66 (40.000)	
TNM stage^a^, no. (%)				0.595
I	1 (0.303)	0 (0.000)	1 (0.606)	
II	9 (2.727)	4 (2.424)	5 (3.030)	
III	104 (31.515)	56 (33.939)	48 (29.091)	
IVa	216 (65.455)	105 (63.637)	111 (67.273)	
Pretreatment cfEBV DNA (copies/ml) (median (range))	935 (0–395000)	825 (0–395000)	940 (0–249000)	0.507
Family history^b^, no. (%)				1
Yes	13 (3.939)	6 (3.636)	7 (4.242)	
No	317 (96.061)	159 (96.364)	158 (95.758)	
Treatment, no. (%)				0.1
IC+CCRT	308 (93.333)	153 (92.727)	155 (93.940)	
CCRT	12 (3.637)	9 (5.455)	3 (1.818)	
IC+RT	10 (3.030)	3 (1.818)	7 (4.242)	

*Abbreviations*: *WHO* World Health Organization, *T* tumor, *N* node, *cell-free EBV DNA* Epstein–Barr virus deoxyribonucleic acid, *IC* induction chemotherapy, *CCRT* concurrent chemoradiotherapy, *RT* radiotherapy

^a^According to the 8th edition of the International Union against Cancer/American Joint Committee on Cancer (UICC/AJCC) staging manual

^b^Family history of nasopharyngeal carcinoma

### Treatment

All patients received radical radiotherapy using an intensity-modulated technique. Target volumes were delineated according to the institutional guidelines that complies with *International Commission on Radiation Units and Measurements Reports 50 and 62* [18, 19]. The prescribed doses were 66–72 grays (Gy)/28–33 fractions to the planning target volume (PTV) of the primary gross tumor volume, 64–70 Gy/28–33 fractions to the PTV of the gross tumor volume of the involved lymph nodes, 60–63 Gy/28–33 fractions to the PTV of the high-risk clinical target volume, and 54–56 Gy/28–33 fractions to the PTV of the low-risk CTV. In this study, of the 330 patients, 308 (93.3%) received IC and CCRT, 12 (3.6%) received IC and RT, and 10 (3.0%) received only CCRT.

The IC regimens comprised one to four cycles of 3-weekly GP, PF, TP, or TPF. The CCRT regimens comprised one to three cycles of 3-weekly cisplatin or nedaplatin. The immunotherapy regimens comprised one to six cycles of 3-weekly camrelizumab, toripalimab, tislelizumab, nivolumab, sintilimab or pembrolizumab. The GP regimen comprised 80 mg/m^2^ cisplatin on day 1 and 1000 mg/m^2^ gemcitabine on day 1 and day 8. The PF regimen comprised 75 mg/m^2^ cisplatin on day 1 and 5-FU 500 mg/m^2^/day continuously from day 1 to day 5. The TP regimen consisted of 75 mg/m^2^ docetaxel on day 1 and 75 mg/m^2^ cisplatin on day 1. The TPF regimen consisted of 60 mg/m^2^ docetaxel on day 1, 60 mg/m^2^ cisplatin on day 1, and 500 mg/m^2^/day 5-FU continuously from day 1 to day 5. The regimens were repeated every 3 weeks for 1–4 cycles. The concurrent chemotherapy regimen consisted of 80–100 mg/m^2^ cisplatin/nedaplatin administered every 3 weeks for a maximum of three cycles, beginning on the first day of RT. The dose of each cycle of immunotherapy was 200 mg for camrelizumab, tislelizumab, sintilimab, and pembrolizumab; 240 mg for toripalimab; and 360 mg for nivolumab.

### Study variables

Thyroid function tests, including thyroid-stimulating hormone (TSH), free T4 (FT4), free T3 (FT3), A-TPO, and thyroglobulin (TG), were measured at the following time points: within 1 month before treatment (before IC), followed every immunotherapy cycle, within 1 month before radiotherapy (before RT), and within 2 months upon radiotherapy completion (after RT).

Thyroid outcomes during treatment were characterized as either (i) normal—normal TSH level and FT4 level within our institutional reference range (TSH: 0.270–4.200 μIU/mL; FT4: 12.000–22.000 pmol/L) throughout treatment—or (ii) thyroid dysfunction—includes hypothyroidism, hyperthyroidism, and biphasic thyroid dysfunction. Hypothyroidism and hyperthyroidism includes clinical type and subclinical type. The clinical hypothyroidism was defined by TSH levels above the upper reference limit with concomitant FT4 levels below the lower reference interval. The subclinical hypothyroidism was defined by TSH levels above the upper reference limit with normal FT4 levels. Clinical hyperthyroidism was defined by TSH levels below the lower reference interval with concomitant FT4 levels above the upper reference interval. Subclinical hyperthyroidism was defined by TSH levels below the lower reference interval with normal FT4 levels. Biphasic thyroid dysfunction was defined by transient hyperthyroidism followed by hypothyroidism. In addition, our institutional reference range for A-TPO is 0–35 U/mL. Patients were considered to have positive A-TPO if their A-TPO was higher than 35 U/mL at any point during surveillance.

Patient demographic and clinical characteristics included gender, age, WHO histology, T stage, N stage, TNM Stage, pretreatment cfEBV DNA, treatment, IC cycles, IC regimens, CCRT cycles, immunotherapy regimens, and immunotherapy cycles. The stage was according to the 8th edition of the American Joint Commission on Cancer (AJCC) staging system. The cfEBV DNA were measured using a real-time quantitative polymerase chain reaction assay amplifying the *Bam*HI-W region of the EBV genome, as previously described [20, 21]. The pretreatment cfEBV was examined within 1 month before treatment and the posttreatment cfEBV was examined within 3 months after radiotherapy.

### Statistical analysis

The data are summarized as frequencies and percentages for categorical variables and as medians and ranges for continuous variables. The chi-square test or Fisher’s exact test was used to compare categorical variables, and the Kruskal–Wallis test was used for continuous variables. Median levels of TSH and anti-thyroid antibody levels were characterized over time. Univariable and multivariable tests of association between thyroid dysfunction and the complete biological response (cBR; defined as undetectable cfEBV DNA) posttreatment were performed using Logistic regression. Patient demographic and clinical characteristics (gender, age, WHO histology, T stage, N stage, TNM Stage, pretreatment EBV DNA, treatment, IC cycles, IC regimens, CCRT cycles, immunotherapy regimens, and immunotherapy cycles) were included as covariates in the multivariable Logistic regression. Tests were two-sided, and *P* values < 0.05 were considered significant. All analyses were performed with R version 3.4.4 (http://www.r-project.org).

## Results

### Patient characteristics

After matching by PSM, a total of 165 pairs were included in the present retrospective analysis. The demographic and clinical characteristics of the immunotherapy and control groups were well matched (Table 1). No statistically significant differences between the two groups were found in the following variables: sex age, pathological type, T stage, N stage, TNM stage, EBV-DNA level, family history, and treatment (Table 1, all *P* values > 0.050). Among the patients, 246 (74.545%) were male, 84 (25.455%) were female, and the median age was 45 years (range, 13–71 years). Most patients (96.970%) had locally advanced nasopharyngeal carcinoma. In addition, 93.333% received IC+CCRT, only 12 patients (3.637%) received CCRT, and 10 patients (3.030%) received IC+RT.

### Incidence of thyroid dysfunctions

**Fig. 1:**
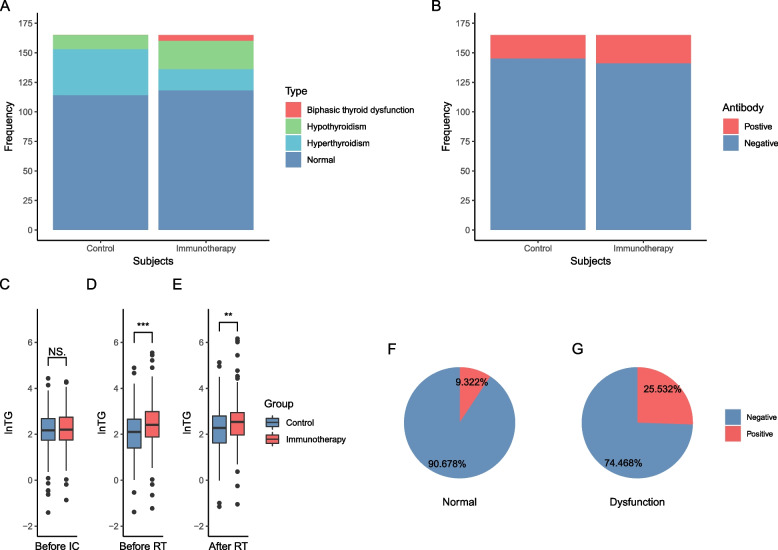
Comparison of the distribution of thyroid dysfunction, positive rates of antithyroid peroxidase antibody, and thyroglobulin levels between the control and immunotherapy groups and the association between thyroid dysfunction and A-TPO. **A** Distribution of thyroid dysfunction in the control and immunotherapy groups. **B** Incidence of A-TPO in the control and immunotherapy groups. **C** Comparison of TG levels before IC between the control and immunotherapy groups. **D** Comparison of TG levels before RT between the control and immunotherapy groups. **D** Comparison of TG levels after RT between the control and immunotherapy groups. **F** Incidence of A-TPO in patients with normal thyroid function in the immunotherapy group. **G** Incidence of A-TPO in patients with thyroid dysfunction in the immunotherapy group. A-TPO, antithyroid peroxidase antibody; TG, thyroglobulin; IC, induction chemotherapy; RT, radiotherapy. NS. *P* > 0.05, **P < 0.01, ***P < 0.001

The spectrum of thyroid dysfunctions was significantly different between the immunotherapy and control groups (Fig. 1A; *P* < 0.001). In the immunotherapy group, 47 patients (47/165, 28.485%) had thyroid dysfunctions, including 24 patients with hypothyroidism (24/47, 51.064%; 11 with clinical type and 13 with subclinical type), 18 with hyperthyroidism (18/47, 38.298%; 8 with clinical type and 10 with subclinical type), and 5 with biphasic thyroid dysfunction (5/47, 10.638%). In the control group, 51 patients (51/165, 30.909%) had thyroid dysfunctions. Among them, 12 patients had hypothyroidism (12/51, 23.529%; 5 with clinical type and 7 with subclinical type), 39 patients had hyperthyroidism (39/51, 76.471%; 7 with clinical type and 32 with subclinical type), and none of the patients had biphasic thyroid dysfunction. The positive rates of A-TPO were similar between the two groups, 12.121% in the control group and 14.545% in the immunotherapy group (*P* = 0.627; Fig. 1B). Before treatment, the level of TG was similar (9.030 in the immunotherapy group (range: 0.102–72.950) vs. 8.500 in the control group (range: 0.135–84.200), *P* = 0.341; Fig. 1C). However, the level of TG increased gradually in the immunotherapy group and was obviously higher than that the control group (before RT: 11.100 (range: 0.293–256) vs. 8.14 (range: 0.046–133.300), *P* < 0.001; after RT: 12.700 (range: 0.350–474.700) vs. 9.710 (range: 0.316–168.500), *P* = 0.008; Fig. 1D, E). Table 2 showed the comparison of onset time and severity of thyroid dysfunctions between the control and immunotherapy group. Compared with the control group, the occurrence of hypothyroidism in the immunotherapy group was earlier with a median onset time difference of 32 days (Table 2, *P* < 0.001). The occurrence time of hyperthyroidism was similar between the two groups (Table 2, *P* = 0.847). No significant difference in the severity of hypothyroidism and hyperthyroidism was identified between the control and immunotherapy group (Table 2, all *P* values > 0.001).

**Table 2:** The median onset time and severity of thyroid dysfunctions between the control and immunotherapy group

		Immunotherapy	Control	*P*
Hypothyroidism	*n*	24	12	
	TSH (mIU/L) (median (range))	5.185 (0.853–48.800)	6.345 (0.608–8.120)	0.341
	FT4 (pmol/L) (median (range))	15.040 (1.230–9.640)	10.860 (9.680–15.620)	0.292
	onset (days) (median (range))	107 (49–137)	139 (100–159)	< 0.001
Hyperthyroidism	*n*	18	39	
	TSH (mIU/L) (median (range))	0.053 (0.018–0.237)	0.069 (0.010–0.465)	0.394
	FT4 (pmol/L) (median (range))	21 (11.500–20.820)	18.27 (13.810–41.100)	0.624
	onset (days) (median (range))	104 (60–148)	112 (27–169)	0.847

*Abbreviations*: *TSH* thyroid-stimulating hormone, *FT4* free T4

### Thyroid functions in patients treated with PD-1 antibody

**Fig. 2:**
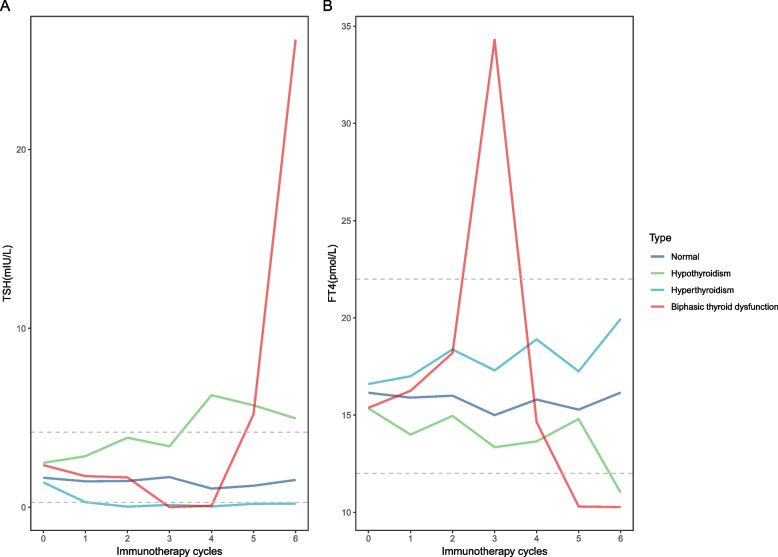
Thyroid-stimulating hormone kinetics and free T4 kinetics during treatment in patients in the immunotherapy group with different patterns of thyroid function. **A** Median TSH during treatment in patients in the immunotherapy group with normal thyroid function, hypothyroidism, hyperthyroidism, and biphasic thyroid dysfunction. Dashed lines represent normal TSH ranges (0.27–4.2 μIU/L). **B** Median FT4 during treatment in patients in the immunotherapy group with normal thyroid function, hypothyroidism, hyperthyroidism, and biphasic thyroid dysfunction. Dashed lines represent normal FT4 ranges (12–22 pmol/L). TSH, thyroid-stimulating hormone; FT4, free T4

The median TSH and FT4 levels over the course of immunotherapy in patients treated with PD-1 antibody are shown in Fig. 2. Individual TSH and FT4 levels are further shown in Additional file 1: Figure S2. In patients with normal thyroid function, their TSH and FT4 levels fluctuated within the normal range (Fig. 2A, B). In patients who developed hypothyroidism, their median TSH level was elevated, and their median FT4 level was decreased outside the normal range. The median number of cycles between the introduction of immunotherapy and the onset of hypothyroidism was 3 (range 1–6 cycles, Fig. 2A, B). The onset of hyperthyroidism was earlier (median 2 cycles, range 1–6 cycles) (Fig. 2A, B). Patients with biphasic thyroid dysfunction first had decreased TSH levels and elevated FT4 levels and then elevated TSH levels and decreased FT4 levels. All of them had lower FT4 levels out of normal range in the hypothyroidic phase (Additional file 1: Figure S2D & H). The median number of immunotherapy cycle was 3 (range 2–4 cycles) between the introduction of immunotherapy and the onset of hyperthyroidism and 5 (range 4–5 cycles) between the introduction of immunotherapy and the onset of hypothyroidism (Fig. 2A, B).

The characteristics of patients treated with PD-1 antibody are presented in Additional file 1: Table S1. No statistically significant differences between the patients with and without thyroid dysfunction were found in the following variables: gender, age, pathological type, T stage, N stage, TNM stage, EBV-DNA level, and treatment-related variables (*P* >0.050).

### A-TPO in patients treated with PD-1 antibody

A-TPO was positive in 12 of 47 patients who developed thyroid dysfunction, compared with 12 of 118 who did not (25.532% vs. 10.169%, *P* = 0.014; Fig. 1F, G). Table 3 shows the incidence of positive A-TPO and the median numbers of immunotherapy cycles before A-TPO onset in patients with normal thyroid function, hypothyroidism, hyperthyroidism, and biphasic thyroid dysfunction. Their median TSH levels, median FT4 levels and median A-TPO levels over the course of immunotherapy are shown in Additional file 1: Figure S3. The individual levels are further shown in Additional file 1: Figure S4. In patients with positive A-TPO and without thyroid dysfunction, the median number of immunotherapy cycles between the introduction of immunotherapy and the presence of A-TPO was 4 (range 1–6 cycles). In patients who had positive A-TPO and developed hyperthyroidism or hypothyroidism, the median cycles before A-TPO onset were 3 (range 1–6 cycles) and 4 (range 2–6 cycles), respectively. Moreover, their A-TPO onset coincided with the onset of thyroid dysfunction. In patients with positive A-TPO and biphasic thyroid dysfunction, the onset of A-TPO (median 2 cycles, range 2–3 cycles) was earlier than that of transient hyperthyroidism.

**Table 3:** Incidence of positive A-TPO and median number of immunotherapy cycles before A-TPO onset

	Normal	Hypothyroidism	Hyperthyroidism	Biphasic thyroid dysfunction	*P*
A-TPO					< 0.001
Positive, no. (%)	12 (10.169)	4 (16.667)	3 (15.000)	5 (100.000)	
Negative, no. (%)	106 (89.831)	20 (83.333)	17 (85.000)	0 (0.000)	
A-TPO onset (cycles) (median (range))	4 (1–6)	4 (1–6)	3 (1–6)	2 (2–63)	0.3403

*Abbreviations*: *A-TPO* antithyroid peroxidase antibody

Additional file 1: Table S1 shows that patients with early-stage disease had higher positive rates of A-TPO than those with advanced-stage disease (50.000% vs. 13.208%, *P* = 0.018). For other demographic and clinical features, no statistically significant differences between the patients with and without A-TPO were found (*P* >0.050).

### Association of immune-related thyroid dysfunction and clinical marker

**Fig. 3:**
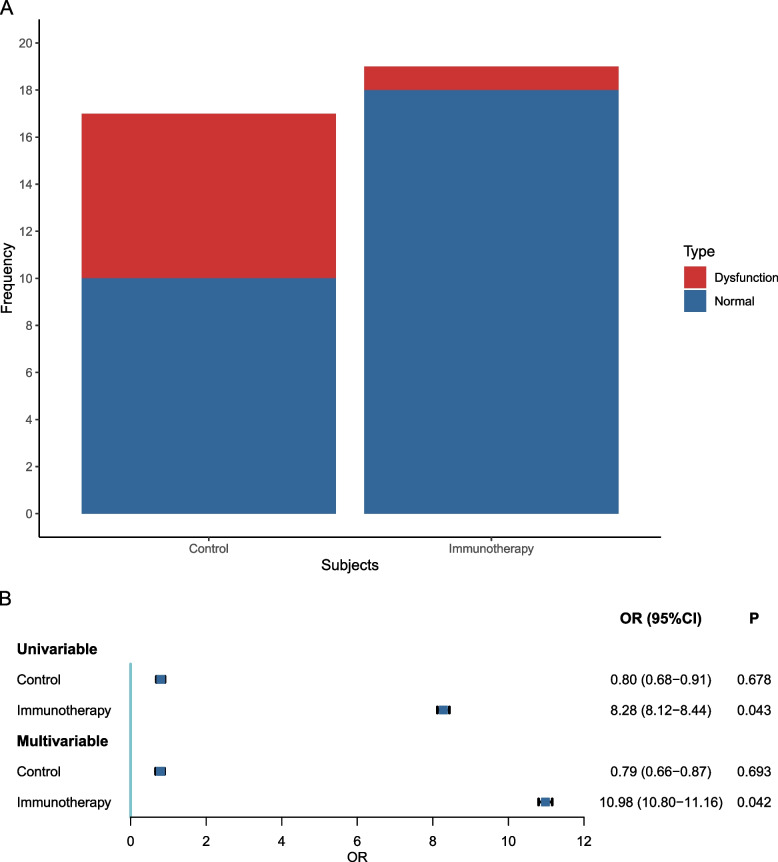
The association between thyroid dysfunction and complete biological response (cBR) within 3 months after treatment. **A** Proportion of thyroid dysfunction in patients with detectable cfEBV DNA within 3 months after treatment in the control and immunotherapy groups. **B** The association between thyroid dysfunction and cBR within 3 months after treatment. The odds ratio (OR) in multivariable Logistic regression was adjusted for patient demographic and clinical characteristics (gender, age, WHO histology, T stage, N stage, TNM Stage, pretreatment EBV DNA, treatment, IC cycles, IC regimens, CCRT cycles, immunotherapy regimens, and immunotherapy cycles)

For nonmetastatic NPC, detectable cfEBV DNA within 3 months after treatment is a clinical marker for predicting disease failure. The cBR to treatment is thought to suggest better survival [21–23]. Within 3 months after treatment, cfEBV DNA was detectable in 17 (10.303%) patients of the control group and in 19 (11.515%) patients of the immunotherapy group (*P* = 0.620). As shown in Fig. 3, 7 of the 17 (41.176%) patients in the control group had thyroid dysfunction (including hypothyroidism, hyperthyroidism, and biphasic thyroid dysfunction). In contrast, only 1 of the 19 (5.263%) patients in the immunotherapy group had thyroid dysfunction (*P* = 0.029). Univariable and multivariable analyses were further performed to evaluate the association between thyroid dysfunction and cBR (Fig. 3). In the control group, no association between thyroid dysfunction and achieving cBR post-treatment was observed (univariable: OR, 0.800; multivariable: OR, 0.790; *P* values > 0.050). In the immunotherapy group, we found an association between thyroid dysfunction and achieving cBR post-treatment (univariable: OR, 8.280; *P* = 0.043), which remained significant after adjusting for patients’ demographic and clinical characteristics (multivariable: OR, 10.980; *P* = 0.042).

## Discussion

This is the first study to explore the incidence and clinical features of thyroid dysfunction and its role as a survival predictor in newly diagnosed, nonmetastatic NPC patients treated with anti-PD-1 immunotherapy. In this study, we found patients in the immunotherapy group developed more hypothyroidism, less hyperthyroidism, and a distinct pattern, biphasic thyroid dysfunction. Immunotherapy also accelerates the development of hypothyroidism. The early elevation of A-TPO without aberrant TSH and FT4 may be a predictive marker for biphasic thyroid dysfunction, a distinct pattern only observed in patients receiving immunotherapy. It is notable that there is an association between the development of thyroid dysfunction and cBR in the immunotherapy group but not in the control group. Therefore, immune-related thyroid dysfunction could be an indicator for better survival but non-immune-related thyroid dysfunction could not.

Our study reported a higher incidence rate of thyroid dysfunction than most other studies. In retrospective studies assessing antibody-mediated thyroid dysfunction, the reported incidence rates were 14.290%, 21%, and 28.380% in non-small-cell lung carcinoma (NSCLC) and 17.780%, 18.180%, and 27.450% in metastatic melanoma. In anti-PD-1 clinical trials for recurrent or metastatic NPC, the range of incidence rates of hypothyroidism during immunotherapy was wide, from 6.700 to 32%. Only one case of hyperthyroidism was reported in the camrelizumab clinical trial for recurrent or metastatic NPC. In anti-PD-1 clinical trials for NSCLC, melanoma, and renal cell carcinoma, the incidence rates of thyroid dysfunction range from 2.500 to 14%, with a mean rate of 5.900% for hypothyroidism and 3.300% for hyperthyroidism [24–29]. The relatively high incidence of thyroid dysfunction in newly diagnosed, nonmetastatic NPC was likely the result of unavoidable irradiation of the thyroid gland during radiotherapy, which may render the thyroid gland more susceptible to autoimmunity [30, 31]. Compared with the control group, the incidence of hyperthyroidism is lower in the immunotherapy group (23.636% vs. 10.909%, *P* = 0.002). As three quarters of hyperthyroidism in the control group was subclinical, the hyperthyroidic effect of radiation during treatment was not strong. The incidences of clinical hyperthyroidism were similar between the two groups (4.242% vs. 4.848%, *P* = 0.792). In the immunotherapy group, thyroid glands suffer damages from both radiation and PD-1 blockade. The incidence of clinical thyroid dysfunction was higher in the immunotherapy group (14.545% vs. 7.273%, *P* = 0.034), which indicates strong damage from PD-1 blockade. Since hypothyroidism is the main form of immune-related thyroid dysfunction, the strong hypothyroidic effect from PD-1 blockade in the immunotherapy group may overwhelm the hyperthyroidic effect of radiation and induce the occurrence of hypothyroidism in vulnerable thyroid glands. Therefore, the immunotherapy group has lower incidence of hyperthyroidism than the control group.

Of note, 3.03% of patients in the immunotherapy group developed a distinct pattern of thyroid dysfunction and transient hyperthyroidism followed by hypothyroidism, which was not observed in the control group. This pattern of thyroid dysfunction resembles thyroiditis and was also reported in patients with other cancers who received anti-PD-1 immunotherapy [13, 15, 32–34]. In this study, we found that all of these patients had positive A-TPO and overt hypothyroidism. In biphasic thyroid dysfunction, the onset of A-TPO was earlier than that of transient hyperthyroidism; however, in hypothyroidism and hyperthyroidism, the onset of A-TPO coincided with thyroid dysfunction. The median number of immunotherapy cycles between the introduction of immunotherapy and the presence of A-TPO in biphasic thyroid dysfunction was 2, earlier than that in normal thyroid function (4 cycles), hypothyroidism (4 cycles), and hyperthyroidism (3 cycles). Therefore, we hypothesized that the early elevation of A-TPO without aberrant TSH and FT4 may be a predictive marker for biphasic thyroid dysfunction.

One of the shortcomings of our study is the limited total duration of follow-up. On the one hand, the relatively short follow-up time hindered the exploration of the relationship between immune-related thyroid dysfunction and survival outcome. Instead, we used cBR, a widely recognized survival marker for NPC, to indirectly explore the relationship between thyroid dysfunction and survival. On the other hand, the incidence rates of thyroid dysfunction and positive rates of A-TPO may be underestimated. In particular, the occurrence of hypothyroidism after hyperthyroidism may be missed since the long-term pattern of thyroid function was not observed. Therefore, in our future study, we will extend the follow-up time to address these questions. Another limitation is that the 3-week interval of surveillance of thyroid function may be too long, and some abnormalities may be missed, especially when the sample size is small. Nevertheless, in our study, the overall changes in thyroid function can still be reflected due to the relatively large sample size. A notable advantage of our study over previous studies is our relatively large sample size [13, 15, 32, 34]. In addition, previous studies have focused on NSCLC and melanoma, in which anti-PD-1 immunotherapy has been extensively used; this is the first study to focus on nonmetastatic NPC, in which anti-PD-1 immunotherapy is still in the clinical trial stage.

## Conclusions

In conclusion, in newly diagnosed, nonmetastatic NPC patients, thyroid dysfunction is common during treatment. Compared with conventional treatment, anti-PD-1 immunotherapy changed the spectrum of thyroid dysfunction and accelerates the onset of hypothyroidism. Acquired thyroid dysfunction is a predictor for better response to immunotherapy but not for routine treatment. Therefore, as a potential predictor for survival, thyroid function should be under regular and intensive surveillance in clinical practice of anti-PD-1 immunotherapy in nonmetastatic NPC patients.

## Supplementary Information

**Additional file 1: Table S1.** Distribution of demographic and clinical characteristics of patients treated with PD-1 antibody. **Figure S1.** Flowchart showing the patient selection process. The medical records of 4003 nonmetastatic NPC patients were screened from an NPC-specific database within the Big Data Intelligence Framework. A total of 165 NPC patients who received conventional treatment and 165 NPC patients who received conventional treatment and anti-PD-1 immunotherapy were selected in a step-wise manner. **Figure S2.** Thyroid-stimulating hormone kinetics, free T4 kinetics and antithyroid peroxidase antibody during treatment in patients in the immunotherapy group with positive and negative antibodies. (A) Median TSH during treatment in patients in the immunotherapy group with positive and negative antibodies. Dashed lines represent normal TSH ranges (0.27–4.2 μIU/L). (B) Median FT4 during treatment in patients in the immunotherapy group with positive and negative antibodies. Dashed lines represent normal FT4 ranges (12–22 pmol/L). (C) Median A-TPO during treatment in patients in the immunotherapy group with positive and negative antibodies. Dashed lines represent normal A-TPO ranges (0–35 U/mL). TSH, thyroid-stimulating hormone; FT4, free T4; A-TPO, antithyroid peroxidase antibody. **Figure S3.** Thyroid-stimulating hormone kinetics and free T4 kinetics during treatment in patients in the immunotherapy group with different patterns of thyroid function. (A) Median and individual TSH levels during treatment in patients in the immunotherapy group with normal thyroid function, (B) hypothyroidism, (C) hyperthyroidism, and (D) biphasic thyroid dysfunction. Dashed lines represent normal TSH ranges (0.27–4.2 μIU/L). (E) Median FT4 during treatment in patients in the immunotherapy group with normal thyroid function, (F) hypothyroidism, (G) hyperthyroidism, and (H) biphasic thyroid dysfunction. Dashed lines represent normal FT4 ranges (12–22 pmol/L). Green, purple, orange, and red lines represent the median level of all patients. Gray lines represent each of the patients. TSH, thyroid-stimulating hormone; FT4, free T4. **Figure S4**. Thyroid-stimulating hormone kinetics and free T4 kinetics during treatment in patients in the immunotherapy group with positive and negative antibodies. (A) Median and individual TSH during treatment in patients in the immunotherapy group with positive antibody and (D) negative antibody. Dashed lines represent normal TSH ranges (0.27–4.2 μIU/L). (B) Median and individual FT4 levels during treatment in patients in the immunotherapy group with positive antibodies and (E) negative antibodies. Dashed lines represent normal FT4 ranges (12–22 pmol/L). (C) Median and individual FT4 levels during treatment in patients in the immunotherapy group with positive antibodies and (F) negative antibodies. Dashed lines represent normal A-TPO ranges (0–35 U/mL). Green, purple, orange, and red lines represent the median level of all patients. Gray lines represent each of the patients. TSH, thyroid-stimulating hormone; FT4, free T4; A-TPO, antithyroid peroxidase antibody.

## Data Availability

The protocol and the statistical code are available on justified request. Under Chinese law and regulations, databases extracted from the Big Data Intelligence Framework at the Sun Yat-sen University Cancer Centre cannot be made publicly available.

## Abbreviations

PD-1: Death protein-1
NPC: Nasopharyngeal carcinoma
IMRT: Intensity-modulated radiotherapy
irAEs: Immune-related adverse events
A-TPO: Anti-thyroid peroxidase
CCRT: Chemoradiotherapy
RT: Radiotherapy
IC: Induction chemotherapy
CCRT: Concurrent chemoradiotherapy
PSM: Propensity score matching
Gy: Grays
PTV: Planning target volume
TSH: Thyroid-stimulating hormone
FT4: Free T4
FT3: Free T3
TG: Thyroglobulin
AJCC: American Joint Commission on Cancer
cBR: Complete biological response
NSCLC: Non-small-cell lung carcinoma
