# Supplementary material for: Thyroid dysfunction in Chinese nasopharyngeal carcinoma after anti-PD-1 therapy and its association with treatment response
Source: BMC Med. 2023 Jan 16;21:18. doi: 10.1186/s12916-022-02697-3 (PMC9843925; doi:10.1186/s12916-022-02697-3)
Supplement: Supplementary file 1 — Additional file 1: Table S1. Distribution of demographic and clinical characteristics of patients treated with PD-1 antibody. Figure S1. Flowchart showing the patient selection process. The medical records of 4003 nonmetastatic NPC patients were screened from an NPC-specific database within the Big Data Intelligence Framework. A total of 165 NPC patients who received conventional treatment and 165 NPC patients who received conventional treatment and anti-PD-1 immunotherapy were selected in a step-wise manner. Figure S2. Thyroid-stimulating hormone kinetics, free T4 kinetics and antithyroid peroxidase antibody during treatment in patients in the immunotherapy group with positive and negative antibodies. (A) Median TSH during treatment in patients in the immunotherapy group with positive and negative antibodies. Dashed lines represent normal TSH ranges (0.27–4.2 μIU/L). (B) Median FT4 during treatment in patients in the immunotherapy group with positive and negative antibodies. Dashed lines represent normal FT4 ranges (12–22 pmol/L). (C) Median A-TPO during treatment in patients in the immunotherapy group with positive and negative antibodies. Dashed lines represent normal A-TPO ranges (0–35 U/mL). TSH, thyroid-stimulating hormone; FT4, free T4; A-TPO, antithyroid peroxidase antibody. Figure S3. Thyroid-stimulating hormone kinetics and free T4 kinetics during treatment in patients in the immunotherapy group with different patterns of thyroid function. (A) Median and individual TSH levels during treatment in patients in the immunotherapy group with normal thyroid function, (B) hypothyroidism, (C) hyperthyroidism, and (D) biphasic thyroid dysfunction. Dashed lines represent normal TSH ranges (0.27–4.2 μIU/L). (E) Median FT4 during treatment in patients in the immunotherapy group with normal thyroid function, (F) hypothyroidism, (G) hyperthyroidism, and (H) biphasic thyroid dysfunction. Dashed lines represent normal FT4 ranges (12–22 pmol/L). Green, purple, orange, and r [file 12916_2022_2697_MOESM1_ESM.docx]

**Additional File 1:**

**Thyroid Dysfunction in Chinese Nasopharyngeal Carcinoma after Anti-PD-1 Therapy Immunotherapy and Its Association with Treatment Response**

**Additional file 1: Table S1**

Table S1. Distribution of demographic and clinical characteristics of patients treated with PD-1 antibody

|  |  | Normal | Thyroid dysfunction | *P* |  | A-TPO negative | A-TPO positive | *P* |
| --- | --- | --- | --- | --- | --- | --- | --- | --- |
| n |  | 118 | 47 |  |  | 141 | 24 |  |
| Gender, No. (%) |  |  |  | 0.921 |  |  |  | 0.902 |
| Male |  | 88 (74.576) | 34 (72.340) |  |  | 105 (74.468) | 17 (70.833) |  |
| Female |  | 30 (25.424) | 13 (27.660) |  |  | 36 (25.532) | 7 (29.167) |  |
| Age (median(range)) |  | 45 (14-71) | 44 (20-68) | 0.657 |  | 44 (14-71) | 49 (25-67) | 0.143 |
| WHO Histology,  No. (%) |  |  |  | 0.913 |  |  |  | 0.848 |
| II |  | 2 (1.695) | 0 (0.000) |  |  | 1 (0.709) | 1 (4.167) |  |
| III |  | 116 (98.305) | 24 (100.000) |  |  | 141 (99.291) | 23 (95.833) |  |
| T stage^a^, No. (%) |  |  |  | 0.108 |  |  |  | 0.014 |
| T1 |  | 4 (3.390) | 4 (8.511) |  |  | 4 (2.837) | 4 (16.667) |  |
| T2 |  | 13 (11.017) | 1 (2.128) |  |  | 11 (7.801) | 3 (12.500) |  |
| T3 |  | 58 (49.153) | 28 (59.574) |  |  | 78 (55.319) | 8 (33.333) |  |
| T4 |  | 43 (36.440) | 14 (29.787) |  |  | 48 (34.043) | 9 (37.500) |  |
| N stage^a^, No. (%) |  |  |  | 0.591 |  |  |  | 0.995 |
| N0 |  | 4 (3.390) | 3 (6.383) |  |  | 6 (4.255) | 1 (4.167) |  |
| N1 |  | 31 (26.271) | 9 (19.149) |  |  | 34 (24.113) | 6 (25.000) |  |
| N2 |  | 35 (29.661) | 17 (36.170) |  |  | 45 (31.915) | 7 (29.166) |  |
| N3 |  | 48 (40.678) | 18 (38.298) |  |  | 56 (39.717) | 10 (41.667) |  |
| TNM Stage^a^,  No. (%) |  |  |  | 0.394 |  |  |  | 0.018 |
| I-II |  | 4 (3.390) | 2 (4.255) |  |  | 3 (2.128) | 3 (12.500) |  |
| III-IVa |  | 114 (96.610) | 45 (95.745) |  |  | 138 (97.872) | 21 (87.500) |  |
| Pretreatment cfEBV DNA (copies/ml) (median(range)) |  | 1003  (0-171000) | 1070  (0-249000) | 0.347 |  | 1125  (0-94000) | 1330  (0-51000) | 0.72 |
| Treatment, No. (%) |  |  |  | 0.687 |  |  |  | 0.77 |
| IC+CCRT |  | 110 (93.220) | 45 (95.745) |  |  | 132 (93.617) | 23 (95.833) |  |
| CCRT |  | 2 (1.695) | 1 (2.128) |  |  | 3 (2.128) | 0 (0.0) |  |
| IC+RT |  | 6 (5.085) | 1 (2.128) |  |  | 6 (4.255) | 1 (4.167) |  |
| IC cycles, No. (%) |  |  |  | 0.741 |  |  |  | 0.737 |
| 0 |  | 2 (1.695) | 1 (2.128) |  |  | 3 (2.128) | 0 (0.0) |  |
| 1 |  | 4 (3.390) | 0 (0.000) |  |  | 4 (2.837) | 0 (0.0) |  |
| 2 |  | 19 (16.102) | 5 (10.638) |  |  | 20 (14.184) | 4 (16.667) |  |
| 3 |  | 79 (66.949) | 34 (72.340) |  |  | 97 (68.794) | 16 (66.666) |  |
| 4 |  | 14 (11.864) | 7 (14.894) |  |  | 17 (12.057) | 4 (16.667) |  |
| IC regimens, No. (%) |  |  |  | 0.703 |  |  |  | 0.823 |
| None |  | 1 (0.848) | 1 (2.128) |  |  | 3 (2.128) | 0 (0.000) |  |
| GP |  | 68 (57.627) | 26 (55.319) |  |  | 78 (55.319) | 15 (62.500) |  |
| PF |  | 3 (2.542) | 1 (2.128) |  |  | 4 (2.837) | 0 (0.0) |  |
| TP |  | 42 (35.593) | 16 (34.042) |  |  | 51 (36.170) | 7 (29.166) |  |
| TPF |  | 4 (3.390) | 3 (6.383) |  |  | 5 (3.546) | 2 (8.334) |  |
| CCRT cycles, No. (%) |  |  |  | 0.123 |  |  |  | 0.986 |
| 0 |  | 5 (4.237) | 1 (2.128) |  |  | 5 (3.546) | 1 (4.167) |  |
| 1 |  | 5 (4.237) | 4 (8.511) |  |  | 8 (5.674) | 1 (4.167) |  |
| 2 |  | 74 (62.712) | 37 (78.723) |  |  | 94 (66.667) | 17 (70.833) |  |
| 3 |  | 33 (27.966) | 5 (10.638) |  |  | 33 (23.403) | 5 (20.833) |  |
| Immunotherapy regimens, No. (%) |  |  |  | 0.386 |  |  |  | 0.419 |
| Camrelizumab |  | 21 (17.797) | 10 (21.277) |  |  | 26 (18.440) | 5 (20.833) |  |
| Nivolumab |  | 3 (2.542) | 2 (4.255) |  |  | 4 (2.837) | 1 (4.167) |  |
| Pembrolizumab |  | 0 (0.000) | 1 (2.128) |  |  | 1 (0.709) | 0 (0.000) |  |
| Sintilimab |  | 51 (43.220) | 14 (29.787) |  |  | 57 (40.426) | 8 (33.333) |  |
| Tislelizumab |  | 9 (7.627) | 3 (6.383) |  |  | 10 (7.092) | 2 (8.334) |  |
| Toripalimab |  | 34 (28.814) | 17 (36.170) |  |  | 43 (30.496) | 8 (33.333) |  |
| Immunotherapy cycles, No. (%) |  |  |  | 0.492 |  |  |  | 0.697 |
| 1-3 |  | 43 (36.441) | 16 (34.043) |  |  | 52 (36.879) | 7 (29.167) |  |
| 4-6 |  | 75 (63.559) | 31 (65.957) |  |  | 89 (63.121) | 17 (70.833) |  |

Abbreviations: WHO, World Health Organization; T, tumor; N, node; cfEBV DNA, cell-free Epstein–Barr virus deoxyribonucleic acid; IC, induction chemotherapy; CCRT, concurrent chemoradiotherapy; RT, radiotherapy; GP, gemcitabine and cisplatin; PF, cisplatin and 5-fluorouracil; TP, docetaxel and cisplatin; TPF, docetaxel, cisplatin, and 5-fluorouracil.

^a^According to the 8th edition of the International Union against Cancer/American Joint Committee on Cancer (UICC/AJCC) staging manual.

**Additional file 1: Figures S1-4**


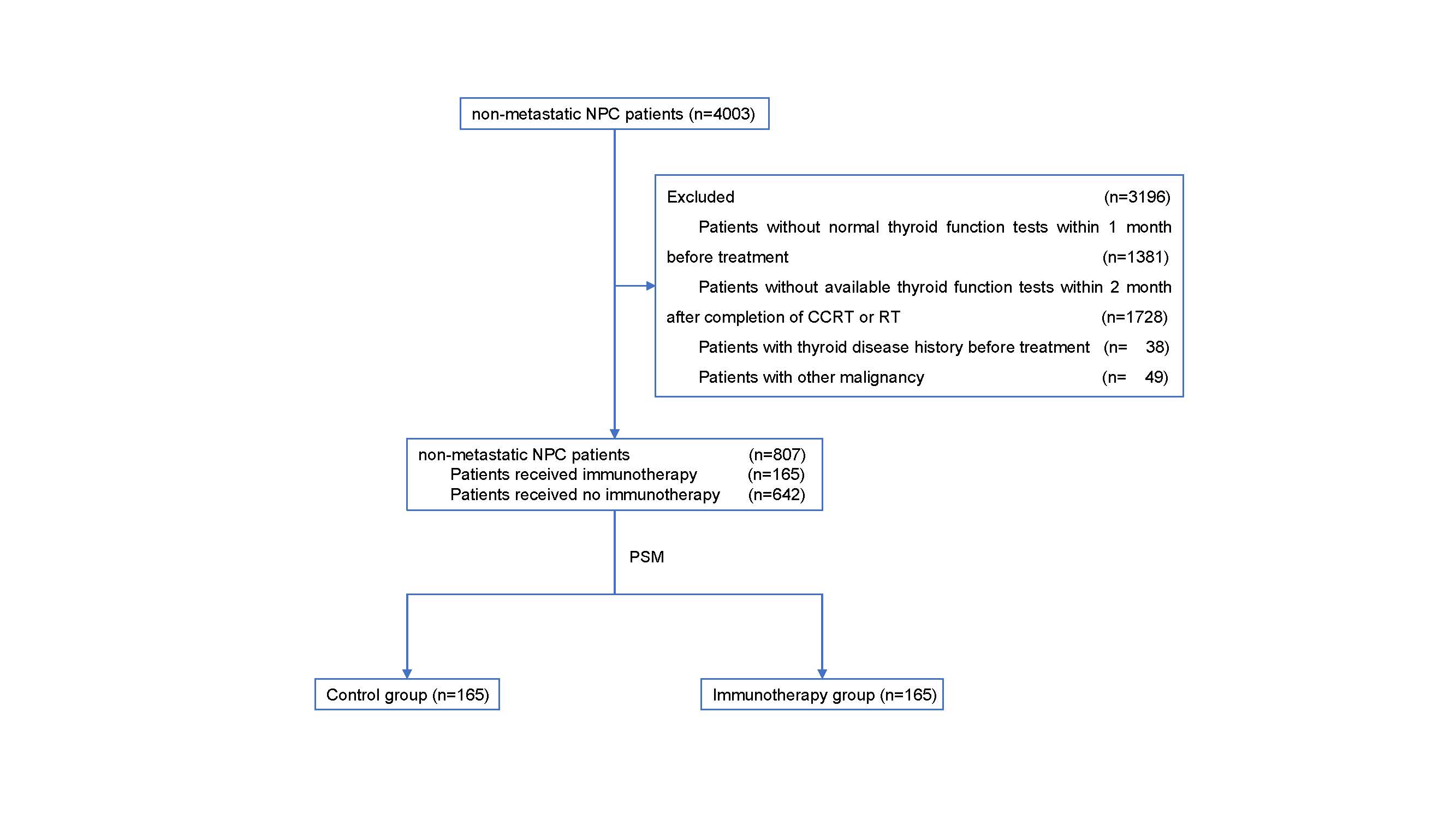


Figure S1. Flowchart showing the patient selection process. The medical records of 4,003 nonmetastatic NPC patients were screened from an NPC-specific database within the Big Data Intelligence Framework. A total of 165 NPC patients who received conventional treatment and 165 NPC patients who received conventional treatment and anti-PD-1 immunotherapy were selected in a step-wise manner.


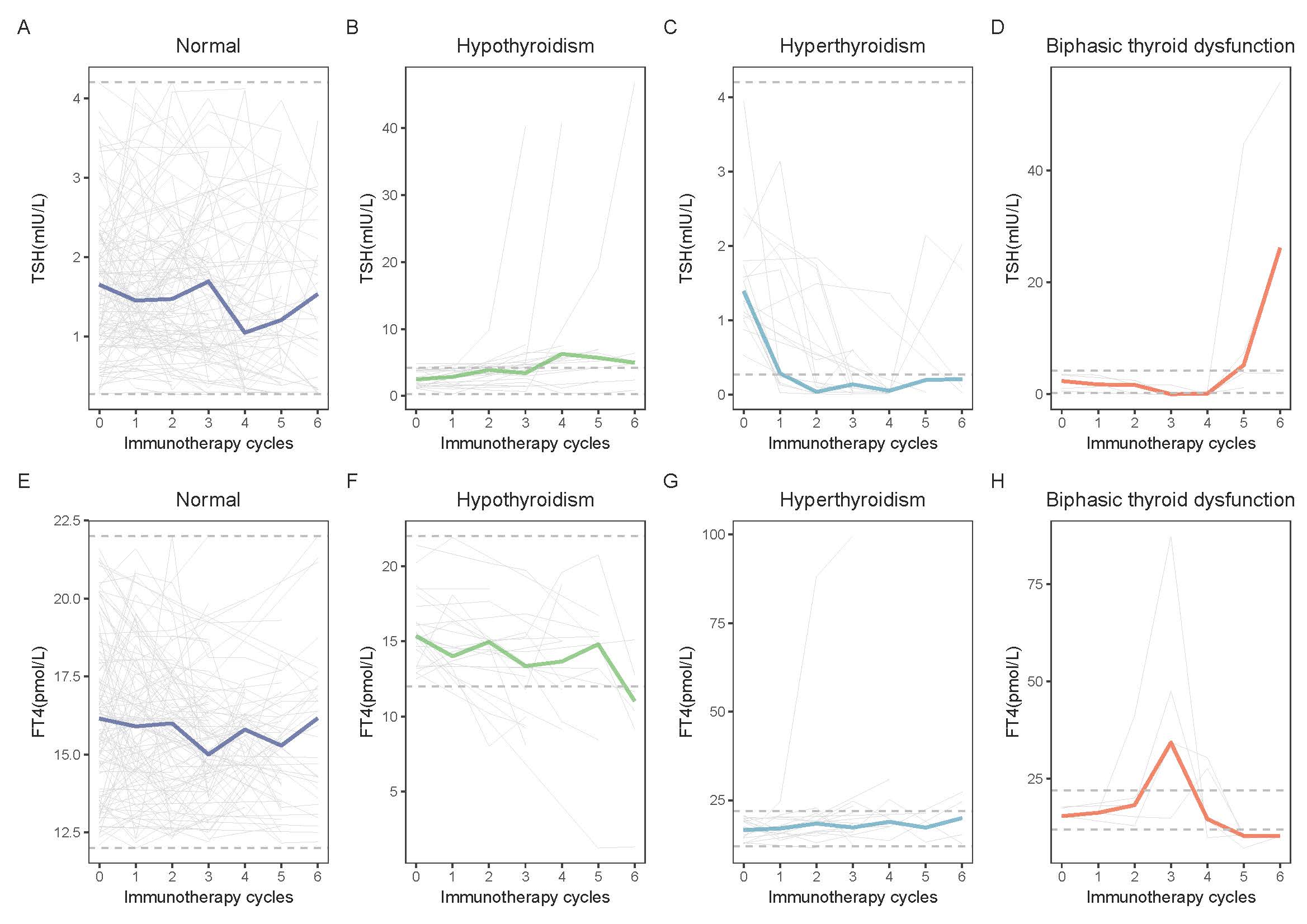


Figure S2. Thyroid-stimulating hormone kinetics, free T4 kinetics and antithyroid peroxidase antibody during treatment in patients in the immunotherapy group with positive and negative antibodies. (A) Median TSH during treatment in patients in the immunotherapy group with positive and negative antibodies. Dashed lines represent normal TSH ranges (0.27–4.2 μIU/L). (B) Median FT4 during treatment in patients in the immunotherapy group with positive and negative antibodies. Dashed lines represent normal FT4 ranges (12–22 pmol/L). (C) Median A-TPO during treatment in patients in the immunotherapy group with positive and negative antibodies. Dashed lines represent normal A-TPO ranges (0–35 U/mL). TSH, thyroid-stimulating hormone; FT4, free T4; A-TPO, antithyroid peroxidase antibody.


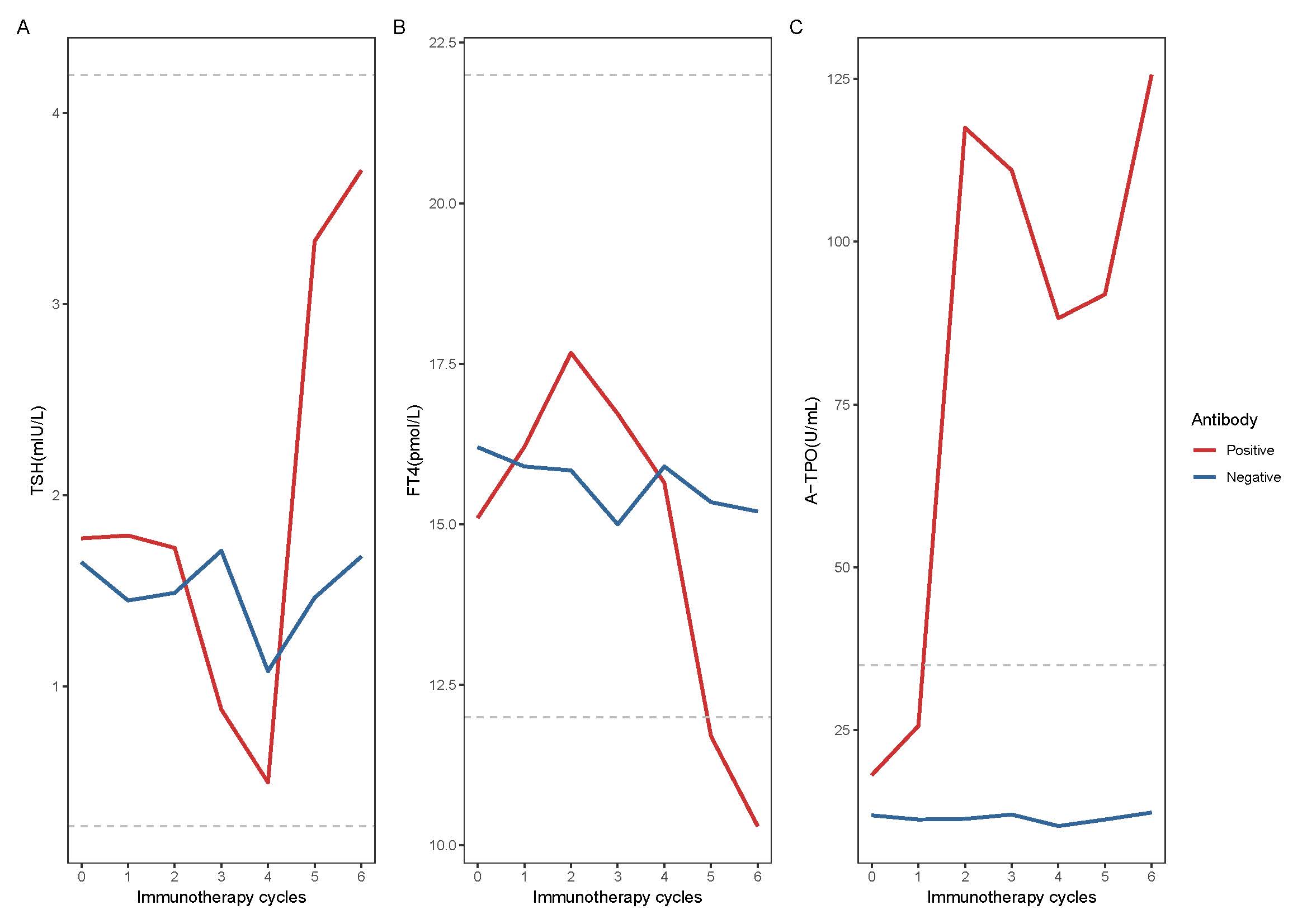


Figure S3. Thyroid-stimulating hormone kinetics and free T4 kinetics during treatment in patients in the immunotherapy group with different patterns of thyroid function. (A) Median and individual TSH levels during treatment in patients in the immunotherapy group with normal thyroid function, (B) hypothyroidism, (C) hyperthyroidism, and (D) biphasic thyroid dysfunction. Dashed lines represent normal TSH ranges (0.27–4.2 μIU/L). (E) Median FT4 during treatment in patients in the immunotherapy group with normal thyroid function, (F) hypothyroidism, (G) hyperthyroidism, and (H) biphasic thyroid dysfunction. Dashed lines represent normal FT4 ranges (12–22 pmol/L). Green, purple, orange, and red lines represent the median level of all patients. Gray lines represent each of the patients. TSH, thyroid-stimulating hormone; FT4, free T4.


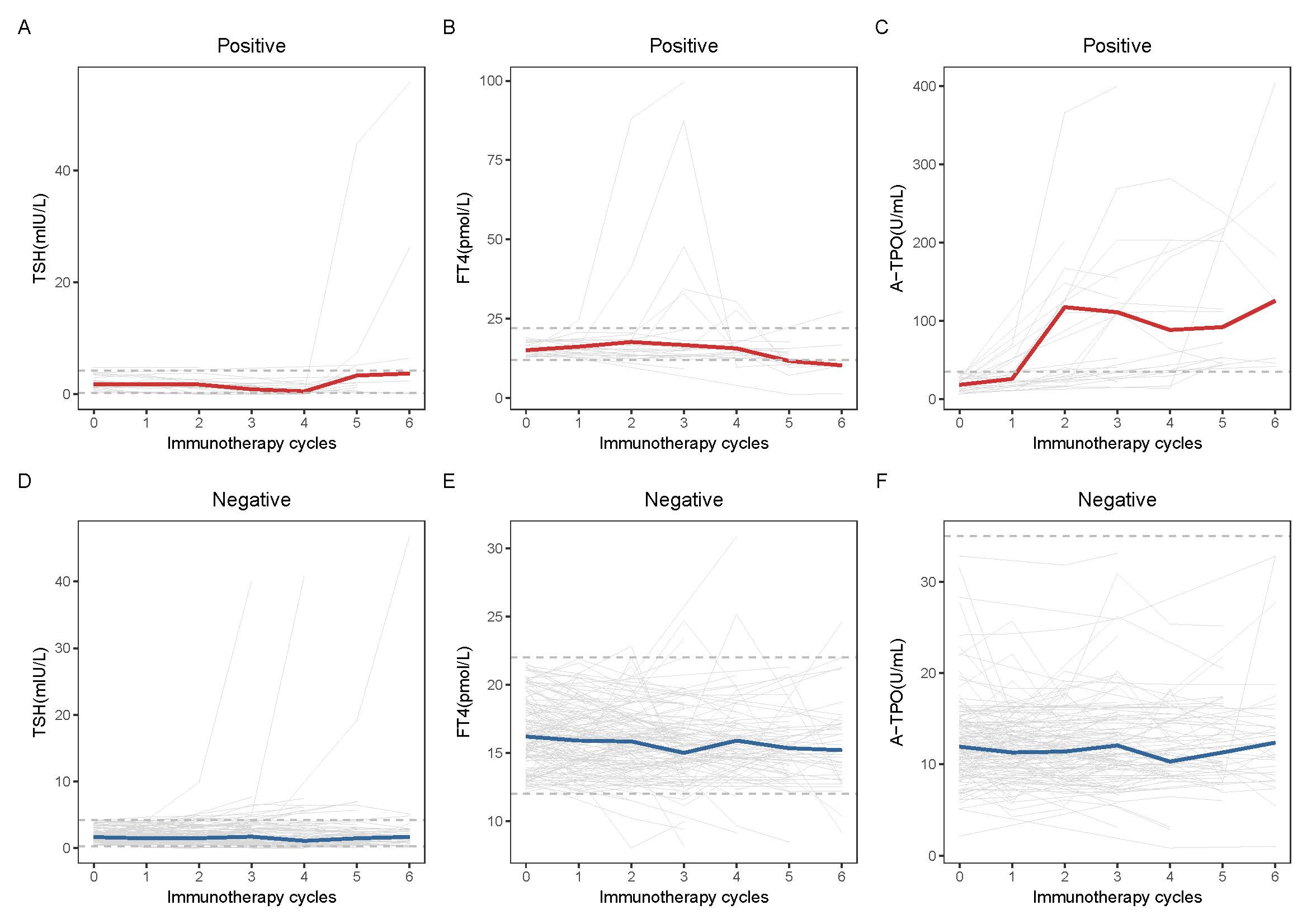


Figure S4. Thyroid-stimulating hormone kinetics and free T4 kinetics during treatment in patients in the immunotherapy group with positive and negative antibodies. (A) Median and individual TSH during treatment in patients in the immunotherapy group with positive antibody and (D) negative antibody. Dashed lines represent normal TSH ranges (0.27–4.2 μIU/L). (B) Median and individual FT4 levels during treatment in patients in the immunotherapy group with positive antibodies and (E) negative antibodies. Dashed lines represent normal FT4 ranges (12–22 pmol/L). (C) Median and individual FT4 levels during treatment in patients in the immunotherapy group with positive antibodies and (F) negative antibodies. Dashed lines represent normal A-TPO ranges (0–35 U/mL). Green, purple, orange, and red lines represent the median level of all patients. Gray lines represent each of the patients. TSH, thyroid-stimulating hormone; FT4, free T4; A-TPO, antithyroid peroxidase antibody.
